# Minimising the usage of desflurane only by education and removal of the vaporisers – a before-and-after-trial

**DOI:** 10.1186/s12871-025-02982-7

**Published:** 2025-02-27

**Authors:** Ferdinand Lehmann, Johannes Mader, Christian Koch, Melanie Markmann, Dominik Leicht, Michael Sander

**Affiliations:** 1 Department of Anaesthesiology, Operative Intensive Care Medicine and Pain Therapy, University Hospital of Giessen, Justus-Liebig University Giessen, Giessen, Germany; 2 Klinik für Anästhesie, operative Intensivmedizin und Schmerztherapie, Krankenhaus im Friedrichshain, Berlin, Germany

**Keywords:** Sustainability, Carbon footprint, General anaesthesia, Desflurane, Awareness of environmental protection

## Abstract

**Background:**

By exceeding planetary environmental boundaries, multiple global crises have become imminent in the 21st century. The healthcare system is a contributor to the climate crisis, accounting for approximately 5% of greenhouse gas emissions in Western countries. In anaesthetic clinics, desflurane, a highly potent greenhouse gas and volatile anaesthetic with no compelling indications, accounts for up to two thirds of total emissions. Its use can be drastically reduced using simple measures. In the present study, we investigated whether a relevant and timely reduction in use could be achieved by dismounting desflurane vaporisers and providing information to the team without restricting its use.

**Methods:**

The study was conducted in a German university hospital with approximately 1250 beds, over a 12-month period between 2021 and 2022, with a comparison to the corresponding periods of the previous years up to 2017. The interventions were, first, the removal of desflurane vaporisers, and second, staff education on the climate impact of volatile anaesthetics. The primary outcome variable was the reduction of hypnotic-related emissions in CO_2_ equivalents per anaesthetic procedure.

**Results:**

Prospective data collection and interventions were conducted from 28 March 2021 to 27 March 2022. The amount of CO_2_ equivalent emissions per procedure in the form of volatile anaesthetics was reduced by 86% compared with the year before the interventions (*p* < 0.001). Interestingly, there was already a 52.1% reduction in the year before the procedure (*p* < 0.001). There were no significant changes in the use of sevoflurane or propofol. Hypnotic-related costs decreased by €14,549, whereas extubation time did not change significantly.

**Conclusions:**

Removal of desflurane vaporisers and staff training can quickly and significantly reduce the emissions of an anaesthesia department in a large German teaching hospital. This may also reduce the costs.

**Trial registration:**

The trial was registered with the German Clinical Trials Register, identifier DRKS00024973 on 12/04/2021.

**Supplementary Information:**

The online version contains supplementary material available at 10.1186/s12871-025-02982-7.

## Background

The anthropogenic climate crisis is one of the most threatening medical issues of the 21st century [1, 2]. The primary cause is the emission of greenhouse gases, including volatile anaesthetic agents (VA) [3]. Of relevance today are isoflurane (Global Warming Potential over 100 years compared to CO_2_ [GWP_100_]) 510, sevoflurane GWP_100_ 130, and desflurane with a GWP_100_ of 2540. Some publications suggest using the global warming potential over 20 years or even 1 year, which are higher for VA in order to emphasise the urgency of climate action [4]. Notably, serious concerns have been raised about the value of GWP as a valid parameter for short-lived trace gases such as VA [5–8]. As the residence time of VA in the tropopause is relatively short compared to CO_2_, a complete distribution in the relevant atmospheric layers is unclear and degradation processes are not sufficiently taken into account. Therefore, the greenhouse effect of VA may be less than assumed. This position is in turn contradicted by other climate researchers, as the processes of how individual greenhouse gases act in the atmosphere have not been clarified in all details but the concept of GWP is a recognised and sensible approximation and is also still used by all relevant institutions for hundreds of substance [9–12]. However, despite their comparatively small impact on overall global warming (less than 0.01% of effective radiative forcing), VA account for up to 2.5% of emissions from the healthcare sector, mainly from nitrous oxide and desflurane [13]. After use of VA, they are generally released into the atmosphere unfiltered via hospital exhaust [14]. As we found in our own department, nitrous oxide, even if used for only a small number of procedures, can contribute significantly to the department’s greenhouse gas emissions with up to 2,140 tonnes CO_2_ equivalent (CO_2_e) per year [15]. Further, desflurane and sevoflurane alone can account for almost 80% of the total emissions of an anaesthesia department [16]. 

A previous study showed that the CO_2_e of two anaesthesia departments in two regional hospitals in Germany could be reduced by 68% by almost completely eliminating desflurane, by dismounting the necessary vaporisers and by educating staff about their carbon footprint [16]. With these simple interventions, education and nudging, the amount of CO_2_e emitted per individual employee was reduced from about 17 to 5 tonnes per year, compared to the national German average of around 11 tonnes per citizen [17]. Another study found that depending on the surgical technique used, VA accounts for more than 90% of the total CO_2_e of an operation [18]. In contrast, propofol has a CO_2_ footprint that is four orders of magnitude lower than that of desflurane [19]. In addition to changing the immediate availability of desflurane, it has been shown that a significant reduction in the CO_2_e of anaesthesia can also be achieved by educating staff and providing information material and educational stickers on the vaporisers [20]. Furthermore, in addition to avoiding the use of desflurane and nitrous oxide, the implementation of sparing techniques for other VAs (sevoflurane and isoflurane) is an additional factor in reducing greenhouse gas emissions and costs. This can be achieved through staff training [21, 22]. Capture systems for desflurane do not appear to sufficiently reduce emissions as yet [23]. 

Desflurane and sevoflurane, the two main modern volatile anaesthetics, have minimal differences in pharmacodynamics and pharmacokinetics. Although desflurane has faster pharmacokinetics and a shorter context-sensitive half-life, it is associated with higher costs [22, 24]. However, studies to date have failed to definitively favour one anaesthetic over the other or over propofol, with some possible advantages for propofol [25–30].

In conclusion, the use of desflurane in anaesthesia practice appears to be dispensable from both an environmental and economic perspective. However, the feasibility of the prompt implementation of effective strategies to reduce the emission of desflurane in a large hospital setting is still uncertain. The aim of this study was to assess the potential for reducing carbon footprint, VA consumption and costs in a large university hospital by limiting the immediate availability of desflurane without compromising its usability. In addition, educating staff about the environmental impact of anaesthesia management is an integral part of our approach.

## Methods

### Setting

This prospective intervention study with a retrospective comparison period was conducted at the Department of Anaesthesiology, Intensive Care Medicine and Pain Therapy, University Hospital Giessen (UKG) in Giessen, Germany with 32 anaesthesia workstations (operating theatres and diagnostics) and approximately 20,000 anaesthetic procedures per year. Ethical approval for this study was obtained from the local Ethics Committee of Justus-Liebig University, Giessen, Germany (Chairperson Prof Dr H Tillmanns) on 22 February 2021, identifier AZ 17/21. The trial was registered with the German Clinical Trials Register (identifier DRKS00024973) and adhered to the latest version of the Declaration of Helsinki [31]. 

Prospective data were collected over a 12-month intervention period between 28 March 2021 and 27 March 2022, and compared with annual data from corresponding retrospective periods from 28 March 2017. Retrospective periods starting from 2017 were chosen because of a relevant change in the medication ordering system at that time. Only data for VA use during anaesthetic procedures was included, excluding the use in intensive care.

Anaesthetic practice was performed according to internal standard operating procedure (SOP). Depth of anaesthesia was controlled by bispectral index or minimum alveolar concentration. Opioids were at the discretion of the anaesthesiologist and are not typically associated with specific VA or propofol.

### Interventions

The first intervention was to remove desflurane vaporisers from immediate availability. Prior to the study, these were standard in almost all anaesthesia workstations. During the intervention phase, the removed vaporisers were stored centrally near the anaesthesia workstations. Remounting was straightforward and included in mandatory equipment training, and did not require consultation with senior physicians or department heads. The warming time of the vaporiser, which takes about ten minutes was considered acceptable.

The second intervention was to inform staff about the environmental impact of volatile anaesthetics. This was achieved through two training sessions, emails via the departmental mailing list, the internal publication of an SOP entitled “Climate-friendly anaesthesia management”, and the placement of information stickers on anaesthesia machines and vaporisers. The training sessions were led by the first author of the study and were open to medical staff and nurses. It included a presentation of the science behind the climate crisis and the contribution of healthcare, followed by advice on how to implement climate-friendly anaesthesia techniques. One session was held online and recorded to make it available to staff who could not attend. The SOP included details of techniques to reduce fresh gas flow (minimal flow) and choice of anaesthetic agents. It was designed by the first author of the study and reviewed by the head of department and senior consultant. Stickers included information on the global warming potential of sevoflurane, as well as QR codes to an app that calculates emissions from the VA, and the German Anaesthesia Society’s 2020 position paper on sustainability.

### Parameters

The primary parameter was the emission of CO_2_e from volatile anaesthetics, proportional to the number of anaesthetic procedures performed over 12 months. The calculation of CO_2_e of VA was based on the GWP_100_ as used in several other articles and the Kyoto Protocol [10, 16, 20, 32]. It was obtained by multiplying the density of the agent by its volume and its specific GWP_100_ [32]. For simplicity, the carbon footprint of propofol was assumed to be zero. Considering its entire life cycle, including disposable plastics for TIVA, it has about 4.5% of the carbon footprint of sevoflurane or about 0.4% of that of desflurane anaesthesia [33]. As this calculation is based on UK data and cannot simply be transferred to German conditions, we did not include propofol in total emission evaluation.

Secondary parameters included the amount and cost of volatile anaesthetics and propofol used, the postoperative time to extubation (emergence time), and the number of vaporiser remounts.

Fresh gas flow could not be determined and compared as it is no standard parameter within our department’s documentation.

### Data collection

The day before the intervention, all vaporisers were refilled, and the remaining stocks of volatile anaesthetic bottles were weighed or counted. These counts were repeated after 1, 6, and 12 months. Data on volatile anaesthetic consumption were collected via order quantities from the UKG central pharmacy and adjusted by the recorded residual stocks. Comparative data for previous years were determined based on the purchase quantities over the corresponding periods. Remounted vaporisers were counted weekly, dismounted, and returned to storage.

The number of anaesthetic procedures performed and emergence times were determined using the department’s internal documentation software (IMESO-IT, Giessen, Germany). Specific data regarding the administering anaesthesiologist, surgical setting, and indications were not collected to prevent individual identification. Emergence time was defined as the interval between the end of the surgical procedure and the patient’s extubation. If extubation occurred before the end of the surgical measures, the duration was recorded as 0 min, in accordance with the typical process sequence. The duration of surgery was defined as the interval between surgical incision and closure of the skin.

### Statistics

Descriptive data are presented as absolute values for each observation period separately, corresponding to whole years. Consumption of VA bottles was pooled monthly to enable statistical comparability. To visualise annual emissions and propofol consumption, box plots with median and interquartile range (IQR) were generated based on pooled monthly data. Depending on the result of the Shapiro-Wilk test, differences between periods regarding the monthly measured consumption rates and calculated CO_2_e emissions, as well as annual averaged emergence time were assessed using parametric (one-factor analysis of variance (ANOVA) and post-hoc Tukey’s HSD test) or non-parametric tests (Kruskal-Wallis test and post-hoc Dunn’s test). Statistical significance was assumed at a two-tailed *p*-value < 0.05.

## Results

Interventions and prospective data collection took place between 28/03/2021 and 27/03/2022. The number of bottles of volatile anaesthetics used including their cost, is shown in Table 1 for the study period together with corresponding retrospective comparison periods from 2017 onwards. Drug costs were obtained from online price lists, as UKG purchase prices are not publicly available (04/2023 prices per bottle: desflurane €126.80, sevoflurane €196.10, isoflurane €164.14, propofol 1%/ 10 ml €7.22, propofol 1%/ 50 ml €12.15, propofol 2%/ 50 ml €23.18) [34]. 

The reduction in desflurane consumption between the intervention period and the previous year was 99% (from 391 to 4 bottles, *p* < 0.001). Interestingly, desflurane consumption decreased by 44.7% in the year prior to the intervention (from 875 to 391 bottles; *p* < 0.001). ANOVA over all periods showed a significant reduction in desflurane use (*p* < 0.001) and isoflurane use (*p* = 0.02). Further, no significant change was observed in the use of propofol or sevoflurane.

The quantity of CO_2_e emissions in the form of volatile anaesthetics is shown in Table 2. The reduction in emissions per anaesthetic procedure in 2020/21 compared to 2021/22 is 86% (*p* < 0.001). The reduction and corresponding effect size of the intervention period to 2019/20 was even greater at 90.8% (*p* < 0.001). Interestingly, there was already a reduction of 52.1% (*p* < 0.001) between 2019/20 and 2020/21.

**Table 1:** Consumption and cost of volatile anaesthetics

Period	Annual consumption in bottles			Cost in Euro			
	Desflurane	Sevoflurane	Isoflurane	Desflurane	Sevoflurane	Isoflurane	Total
2017/18	852	985	118	108,034	193,159	19,369	320,561
2018/19	863	839	100	109,428	164,527	16,414	290,370
2019/20	875	835	61	110,950	163,744	10,013	284,706
2020/21	391	845	50	49,579	165,705	8,207	223,490
2021/22	4	986	30	507	193,355	4,924	198,786

Data are shown as absolute numbers

**Table 2:** Emissions of volatile anaesthetics as CO_2_ equivalents in relation to number of performed anaesthetic procedures

Period	CO_2_e [t] from				Anaesthetic procedures [n]	CO_2_e per anaesthetic procedure
	Des- flurane	Sevo- flurane	Iso- flurane	Total		
2017/18	760,891	46,713	22,041	829,644	21,815	38.03
2018/19	770,714	39,789	18,679	829,182	22,299	37.18
2019/20	781,431	39,599	11,394	832,424	21,516	38.69
2020/21	349,188	40,073	9,339	398,600	19,736	21.00
2021/22	3,572	46,760	5,604	55,935	20,936	2.67

Data are shown as absolute numbers. CO_2_e – CO_2_ equivalents

**Fig. 1:**
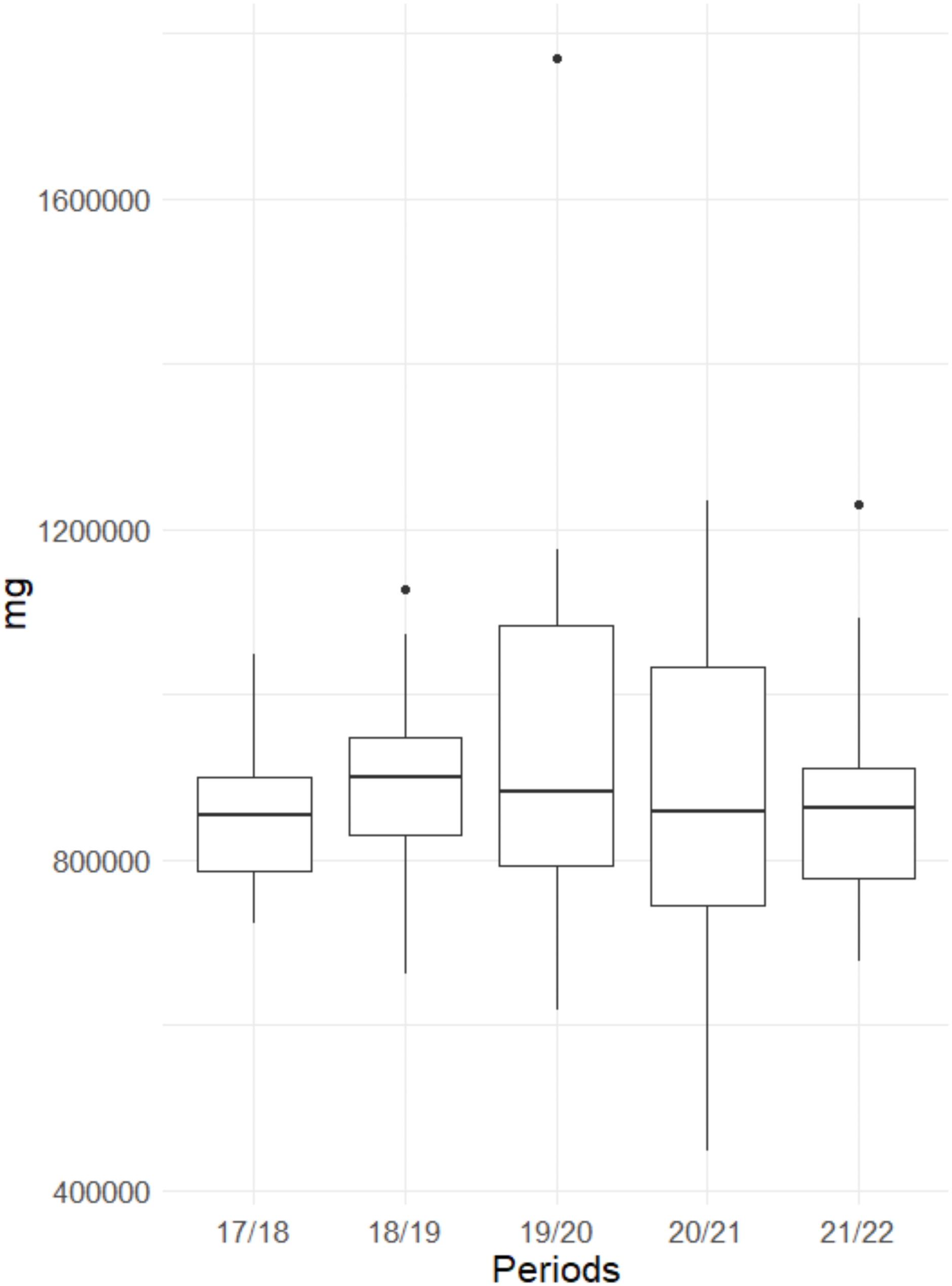
Consumption of propofol over the study periods. Data shown as box plot. Each box is based on monthly consumption of Propofol (12 data points per box). Propofol amount in milligram [mg]

Table 3; Fig. 1 show the consumption and cost of propofol, comparing the study period with the corresponding previous years. The total annual cost of anaesthetics decreased by €14,549 during the study period.

**Table 3:** Consumption and cost of Propofol

Period	Propofol in mg	Cost in Euro
2017/18	10,285,000	288,103
2018/19	10,740,000	299,288
2019/20	11,531,000	318,598
2020/21	10,255,000	277,162
2021/22	10,556,000	287,317

Data are shown as absolute numbers

The time elapsed between the end of the surgical procedure and extubation averaged over the study period is shown in Table 4. The Shapiro-Wilk test showed a non-normal distribution. The subsequent Kruskal-Wallis test showed significant changes over the study period for all procedures (*p* < 0.001). Detailed analysis using Dunn’s test showed significant changes between period 2020/21 and all other periods (all < 0.01). However, no other significant changes were found compared to the intervention period 2021/22. There were no missing data points. The duration of surgery changed over the course of the study (Kruskal-Wallis test *p* < 0,001), but not when comparing the year of intervention with the period before (Dunn’s Test *p* = 0,16).

**Table 4:** – Time in minutes between end of surgical procedures and extubation. Duration of surgery in minutes

Period	All procedures	GA	GA TIVA	GA with VA	GA Desflurane	GA Sevoflurane	GA Isoflurane	Duration of surgery
2017/18	2 (6)	3 (7)	0 (3)	4 (7)	4 (5)	5 (7)	4 (6)	59 (86)
2018/19	2 (6)	4 (7)	0 (4)	5 (7)	4 (6)	5 (7)	5 (6)	60 (87)
2019/20	1 (6)	4 (7)	0 (3)	5 (7)	4 (5)	6 (7)	4 (7)	60 (87)
2020/21	1 (6)	4 (8)	0 (3)	5 (8)	4 (5)	5 (7)	5 (4.75)	63 (92)
2021/22	1 (6)	4 (8)	0 (4)	5 (7)	3 (2.5)	5 (7)	5 (6)	63 (87)

Data are shown as median (interquartile range) in minutes. GA – general anaesthesia, TIVA – total intravenous anaesthesia, VA – volatile anaesthetic

## Discussion

Desflurane elimination is a highly effective strategy for reducing greenhouse gas emissions from anaesthesiology departments. To the best of our knowledge, this study is the first to demonstrate that almost complete cessation of desflurane use in a large university hospital can be achieved in a short period of time through education and changes in drug availability without restriction of the drug. As a result, the total emissions from volatile anaesthetics (excluding nitrous oxide) in our anaesthesiology department decreased by 80% to > 90%. In the Anthropocene with the onset of the climate crisis, we need to find timely ways to reduce greenhouse gas emissions and to adapt to the resulting health threats without compromising the quality of medical care. Anaesthesiology departments have great opportunities in order to facilitate this change [35]. The results of this study underscore the potential for significant timely environmental improvements in healthcare systems.

Our results are consistent with those of Richter et al., who observed a 94.5% reduction in desflurane use in two smaller German hospitals by removing desflurane vaporisers and information dissemination [16]. Similarly, an Australian study reported a 96% reduction in desflurane emissions and an 88% reduction in combined desflurane and sevoflurane emissions through similar measures, but over a longer period [20]. Our study shows that such measures can be implemented rapidly, which is crucial given the urgency of the climate crisis. Another German study showed that a 72% reduction of desflurane use was achievable with educational interventions alone (stickers, posters, presentation) [36]. Compared to our study, a higher reduction in desflurane use > 99% may be due to unmounted desflurane vaporisers.

Interestingly, there was already a highly significant reduction in greenhouse gas emissions in the year before the intervention, due to the reduced use of desflurane. Although this reduction (52.1%) was less than that achieved during the intervention (86%), it still indicates a positive trend in anaesthesiology practice. This shift may be influenced by increasing awareness of the environmental impact of volatile anaesthetics, as highlighted by the 2020 position paper on sustainability by German anaesthesiology societies and the ecological initiatives of the British National Health Service (NHS) [37, 38]. Although the use of desflurane was halved prior to the study, the almost complete cessation of desflurane after the start of the interventions indicates its effect. In contrast, it is possible that the prior decrease marked some kind of priming that was necessary for the intervention to be effective. However, other research suggests that the removal of vaporisers and staff training have the potential to reduce desflurane use substantially, as in our study. Further research is needed to distinguish between the effects of interventions and climate awareness.

In addition to the reduction in emissions, the cost of volatile anaesthetics decreased, mainly due to the increased use of sevoflurane, which requires smaller amounts to achieve the desired anaesthetic effect [39]. The amount of propofol used did not change significantly, resulting in a total cost reduction of €14,549 compared to the previous year. This cost saving is likely to be conservative, as the number of anaesthetic procedures and operations was reduced during the 2020/2021 pandemic period, similar to other hospitals in Germany [40]. In addition to the market price of drugs, the socio-economic consequential costs of greenhouse gas emissions should be considered. Given the current cost estimate of €809 per tonne of CO_2_ equivalent calculated by the German Federal Environment Agency, internalising these costs via emission taxation would further enhance the economic benefits of the emission savings achieved in this study [41]. 

Several additional strategies to reduce desflurane-related emissions are possible. The European Union is considering a complete ban on desflurane, similar to the NHS decommissioning that took effect in 2024. While this may face opposition from anaesthesiology societies, providing comprehensive information on the environmental impact of desflurane, pricing emissions and technical solutions such as filtering anaesthetics are viable options. However, the latter may face supply chain challenges and capture only a fraction of emissions.

The literature suggests that the pharmacological advantages of desflurane, such as better controllability due to a lower blood-gas partition coefficient, could affect emergence times and workflow efficiency [24, 42–44]. However, our study found only significant changes in emergence times during the peak pandemic period 2020/21 compared with all other years. No significant change was found when comparing the intervention period with previous non-pandemic years. This suggests that the intervention had no relevant impact on overall perioperative workflow. Interestingly, over the period of our study, a gradual increase in emergence times was observed for all procedures under general anaesthesia, probably reflecting the ageing and increasing morbidity of the patient population in Germany [45]. The occasional use of desflurane and isoflurane during the intervention period was mostly limited to special cases with particularly long or short extubation times such as adult cardiac surgery for isoflurane and certain paediatric cardiac surgery for desflurane; however, these were rare and did not significantly affect the overall workflow (Table 4). Shorter extubation times with TIVA are most likely due to the typical procedures in which propofol was used in our department, such as general anaesthesia for diagnostic purposes or short surgical procedures.

### Limitations

The study design, which included a prospective intervention with a retrospective comparison group, had several advantages and disadvantages typical of real-world data. A major limitation is the lack of a randomised control group, which makes it difficult to disentangle the effects of the intervention from other variables such as increasing climate awareness. Awareness of the upcoming trial within our department may have contributed to the early changes in practice. In order to avoid bias, comprehensive, evidence-based, unbiased education was provided to all employees. In addition, the choice of anaesthetic was left to each individual. Nevertheless, the substantial impact of the intervention suggests that proactive measures are essential for significant environmental change. Further, the immediate discontinuation of desflurane demonstrates the potential of the intervention to achieve rapid and substantial emission reductions, which are essential for ecological transformation. Comparisons with recent years, particularly the significant but smaller reduction in the year before the trial, highlight the impact of the intervention despite these limitations.

The amount of propofol and sevoflurane used did not change significantly during intervention period. The most likely explanation is the increased use of minimal fresh gas flow, but this cannot be proven due to a lack of data. Fresh gas flow is not part of the standard documentation, so could not be assessed after the intervention - another limitation of the study.

Our calculation of the climate impact was based on the GWP_100_, whereas there is discussion about using 1-year or 20-year global warming potentials, which are much higher [4]. This may emphasise the urgency of climate action, but may also reduce comparability with other studies [16]. 

In addition, the use of GWP itself to quantify VA has been questioned, as it simplifies complex climate processes and may overestimate the impact of short-lived trace gases [6]. Nevertheless, we have chosen the GWP metric over effective radiative forcing as it makes our study comparable to others and facilitates the assessment of multiple emissions. In addition, our aim is to demonstrate the potential for small changes in anaesthetic practice rather than to accurately assess the reduction in carbon emissions. This simplification of quantification also excluded smaller factors in carbon emissions, such as the life cycle of products or energy use. In addition, GWP is suggested by the Intergovernmental Panel on Climate Change when its significant limitations are taken into account [46]. 

It is also questionable whether the impact of discontinuing VA is worth the effort of climate action such as in our study. Valid concerns have been raised regarding the translation of VA emissions into impact on climate change as their effect is minimal (< 0.01% of total greenhouse gas emissions) [5–8]. Only desflurane, with an atmospheric lifetime of 14 years, may have a relevant radiative effect, which again is minimal due to its concentration. Nevertheless, we believe that the choice of short-lived agents (such as sevoflurane) or propofol and minimal flow techniques are quick and easy to implement to reduce our own contribution to climate change, without compromising the quality of care [47]. Moreover, this relatively simple climate action may inspire teams to implement further measures to reduce the carbon emissions of medical departments.

Drug consumption was estimated using pharmacy purchase data, which may not account for unused or wasted VA bottles. To mitigate this, we conducted stock counts at the baseline, at one month, six months, and 12 months to minimise the risk of inaccuracy. However, this approach avoids the complexities of directly quantifying anaesthetic consumption using anaesthesia machines or documentation.

The availability of desflurane changed significantly in our study. Although it was still available within a short time, this is still a strong incentive not to use it. Some anaesthesiologists may interpret this as a near ban. However, the drug was still available and at the discretion of the anaesthesiologists. Other drugs, such as opioids, which are stored in a safe near the workplace, may take even longer to prepare for use. The necessary warming of the vaporiser does not alter care when desflurane use is adequately anticipated and did not cause any issues within our intervention period. However, the removal of desflurane vaporisers from immediate availability is a potential bias in the choice of appropriate drugs.

This study simplified the hypnotic cost calculations. A detailed cost analysis would require additional parameters, including disposable materials for TIVA, the electricity costs for infusion pumps and vaporisers, maintenance costs, real purchase costs, and waste disposal costs. In addition, the high socio-economic costs of greenhouse gas emissions are difficult to quantify accurately, justifying the simplified cost analysis used in this study. Furthermore, the calculation of emissions is simplified by not considering propofol and TIVA materials, as they are minimal compared to VA and difficult to assess accurately for different health care systems.

Notably, as we used aggregated data for the analysis, the validity of the evaluation of emergence time is limited. The complexity of assessing emergence times which is evaluated in specially designed studies, cannot be sufficiently evaluated with our trial. Kruskal-Wallis testing was performed to detect major alteration, which we did not find.

The COVID-19 pandemic is likely to have introduced biases due to changes in healthcare routines. To address this, we correlated the primary outcome with the number of anaesthetic procedures, recognising that surgical capacity was temporarily limited. We also assessed the duration of surgery to rule out major changes in surgical practice between the year of surgery and the previous year. Changes in the type of surgery and patient population were not considered, as this was beyond the scope of this study. Also, changes in the post-extubation period could not sufficiently be evaluated as the pandemic altered care in too many aspects. The modest and statistically insignificant decrease in procedures in the year before the intervention suggests that the pandemic had a limited effect on the primary parameter of the study. In addition, it is very difficult to assess clinical outcome variables in a pandemic setting with an intervention such as this study. Also, only short-term changes in clinical parameters may be expected when desflurane is replaced by propofol or sevoflurane. Therefore, we did not evaluate clinical outcome parameters in our study.

## Conclusion

Emission reduction measures must be implemented swiftly in response to the imminent climate crisis. Unmounting desflurane vaporisers and educating staff can rapidly and significantly reduce emissions in anaesthesiology departments of large teaching hospitals. This approach also offers cost savings and does not adversely affect emergence times. Further research is needed to reduce the carbon footprint of healthcare systems, particularly in developed countries.

## Electronic supplementary material

Below is the link to the electronic supplementary material.

Supplementary Material 1

## Data Availability

The datasets used and/or analysed during the current study are available from the corresponding author upon reasonable request.

## Declarations

### Statements

This study adheres to CONSORT guidelines.

## Abbreviations

ANOVA: Analysis of variance
CO_2_e: CO_2_ equivalent
GWP_100_: Global warming potential over 100 years
IQR: Interquartile range
NHS: National health service
SOP: Standard operating procedure
TIVA: Total intravenous anaesthesia
UKG: University hospital gießen
VA: Volatile anaesthetics
